# The Role of Obesity in the Association Between Alcohol Consumption and HDL‐c Levels: Baependi Heart Study

**DOI:** 10.1002/lipd.70050

**Published:** 2026-03-14

**Authors:** Larissa Esthefani Barros Cirino, Divanei Zaniqueli, Oscar Geovanny Enriquez‐Martinez, Camila Maciel de Oliveira, Alexandre da Costa Pereira, Jordana Herzog Siqueira, Rafael de Oliveira Alvim

**Affiliations:** ^1^ Graduate Program in Health Sciences Federal University of Amazonas Manaus Amazonas Brazil; ^2^ Clinic of Cardiovascular Investigation Federal University of Espírito Santo (UFES) Vitória Espírito Santo Brazil; ^3^ Graduate Program in Public Health Federal University of Espírito Santo Vitória Espírito Santo Brazil; ^4^ Laboratory of Genetics and Molecular Cardiology, Heart Institute University of São Paulo (InCor‐FMUSP) São Paulo SP Brazil; ^5^ Leônidas and Maria Deane Institute/Oswaldo Cruz Foundation Manaus Amazonas Brazil; ^6^ Department of Physiological Sciences Federal University of Amazonas Manaus Amazonas Brazil

**Keywords:** alcohol consumption, cardiovascular diseases, HDL cholesterol, lipid profile, obesity

## Abstract

Cardiovascular diseases (CVD) are a leading cause of global mortality, with dyslipidemia playing a central role in their pathogenesis. The influence of alcohol consumption on lipid profiles, particularly high‐density lipoprotein cholesterol (HDL‐c), in relation to obesity status remains insufficiently explored. We evaluated the association between alcohol consumption and HDL‐c levels in individuals with and without obesity in a Brazilian population. This cross‐sectional analysis used data from the Baependi Heart Study, comprising 2345 participants aged 18–100 years. Alcohol intake was categorized according to weekly ethanol consumption, and HDL‐c levels were measured through standard biochemical methods. No significant differences were observed across alcohol consumption groups for total cholesterol, LDL‐c, triacylglycerols, and fasting glucose; however, the HDL‐c/LDL‐c ratio was significantly higher among male moderate consumers. A significant interaction was found between obesity and moderate alcohol consumption (*β* = 0.90, *p* = 0.015), indicating that the relationship between alcohol intake and low HDL‐c varies according to obesity status. Moderate alcohol consumers exhibited significantly higher HDL‐c levels compared to abstainers, an association observed exclusively among nonobese participants. In this group, moderate alcohol intake was linked to a 65% reduction in the odds of low HDL‐c in women and a 66% reduction in men. No significant association was observed among individuals with obesity. Moderate alcohol consumption was associated with higher HDL‐c levels and lower odds of low HDL‐c, particularly among individuals without obesity. These findings contribute to the understanding of the complex interplay between alcohol intake, lipid metabolism, and adiposity.

**Abbreviations**
ABCA1: ATP‐binding cassette transporter A1
ABCG1: ATP‐binding cassette transporter G1
AC1: first category
AC2: second category
ApoAI: apolipoprotein A‐I
BMI: body mass index
BP: blood pressure
CVD: cardiovascular diseases
DBP: diastolic blood pressure
DM: diabetes mellitus
ELSA‐BRASIL: Longitudinal Study of Adult Health—Brazil
HbA1c: glycated hemoglobin A1C
HDL‐c: high‐density lipoprotein cholesterol
HPLC: high‐performance liquid chromatography
IDL‐c: intermediate‐density lipoprotein cholesterol
IPAQ‐SF: international physical activity questionnaire—short form
LDL‐c: low‐density lipoprotein cholesterol
LPL: lipoprotein lipase
SBP: systolic blood pressure

## 1 Introduction

Cardiovascular diseases (CVD) are the leading cause of death worldwide, accounting for almost 19 million deaths annually, representing 33% of all deaths globally (WHO 2021; Di Cesare et al. 2024). Among the modifiable risk factors for CVD, dyslipidemias play a significant role due to their direct association with the development of atherosclerosis (Catapano et al. 2016). Notably, a significant association exists between low levels of high‐density lipoprotein cholesterol (HDL‐c) and an increased cardiovascular risk (Zhang et al. 2012). The underlying mechanism might involve the role of HDL‐c in reverse cholesterol transport, along with its recognized anti‐inflammatory, antioxidant, and endothelial protective properties (Rader and Hovingh 2014; Mineo and Shaul 2012). The most prominent factors associated with low HDL‐c levels include metabolic conditions such as obesity (Rashid and Genest 2007), insulin resistance (Medeiros et al. 2011), and hypertriglyceridemia (Espondaburu 2006), as well as lifestyle‐related factors such as inadequate diet (Cervato 1997) and excessive alcohol consumption (Albert et al. 2003).

The relationship between alcohol consumption and cardiovascular risk has been extensively examined, with evidence suggesting that light to moderate intake may be linked to a lower risk of CVD (Albert et al. 2003; Constant 1997; Iijima et al. 2002). Of particular interest is the effect of alcohol on lipid metabolism, as moderate consumption has been shown to improve lipid profiles, primarily by increasing HDL‐c levels. This association is supported by several meta‐analyses, which demonstrate that moderate intake of ethanol—including specific beverages like wine and beer—consistently elevates HDL‐c and apolipoprotein concentrations (Rimm et al. 1999; Brien et al. 2011; Huang et al. 2017; Spaggari et al. 2020; Lucerón‐Lucas‐Torres et al. 2025; Khatiwada et al. 2025). However, recent findings indicate that excessive alcohol consumption is associated with markedly elevated HDL‐c levels (Enriquez‐Martinez et al. 2023), which may, paradoxically, increase the risk of cardiovascular mortality (Mamede et al. 2024). It is important to note that the relationship between alcohol consumption and HDL‐c concentrations is modulated by multiple factors, including genetic predisposition (Mach et al. 2020; Leança et al. 2010), metabolic status (Klop et al. 2013), and body weight (Martínez‐Urbistondo et al. 2024).

Nevertheless, studies exploring this relationship among individuals with different nutritional statuses, particularly those with and without obesity, remain scarce. Therefore, the present study aims to evaluate the association between alcohol consumption and HDL‐c levels in individuals with and without obesity in a Brazilian sample. Since obesity is associated with reduced HDL‐c, we hypothesize that excess body weight mitigates the relationship between alcohol consumption and HDL‐c levels.

## 2 Materials and Methods

### 2.1 Study Design and Population

The Baependi Heart Study is a longitudinal cohort study primarily aimed at exploring the genetic factors underlying CVDs in a predominantly rural Brazilian population. More comprehensive details about the study's characterization and methodologies were provided in a previous publication (Egan et al. 2016). Baependi is a small town in Minas Gerais, southeastern Brazil, with an area of 751 km^2^ and a population of 18,366 inhabitants (IBGE 2022). Despite its rural setting, about 70% of Baependi's inhabitants reside in the urban area. The region's economic activity does not revolve around agriculture or livestock, mainly due to the rugged terrain. Instead, the economy is driven by religious tourism and the sale of handcrafted goods.

For this specific analysis, a cross‐sectional study was conducted using data from the second follow‐up visit (2010–2013), which included 2345 participants aged 18–100 years from 109 families residing in the region. Probands were identified from the community at large in several stages. First, 11 census districts (from a total of 12) were selected for study. Second, residential addresses within each district were randomly selected (first by randomly selecting a street, second a household). Finally, eligibility criteria (any individual living in the selected household who was 18 years old or above) within each household were established. Once a proband was enrolled, all his/her first‐degree (e.g., parents, siblings, and offspring), second‐degree (e.g., halfsiblings, grandparents/grandchildren, aunts/uncles, nieces/nephews, and double cousins), and third‐degree (e.g., first cousins, great‐uncles/great‐aunts, and greatnephews/great‐nieces) relatives and his/her respective spouse's relatives, who were at least 18 years old, were invited to participate. After the proband's first contact, first degree relatives were invited to participate by phone; these included all living relatives in the city of Baependi (urban and rural area) and surrounding cities. To recruit the participants, the project was advertised through provincial, religious, and municipal authorities, in local television, newspaper, and radio messages, through physicians, and by phone calls. For physical examination, a clinic was established in a quiet but easily accessible sector of Baependi. Regarding exclusion criteria, individuals with physical or mental disabilities that prevented travel to the research clinic or the completion of the study protocol were excluded. Additionally, pregnant women were excluded from the study. These criteria were established to ensure the safety of the participants and the reliability of the clinical and physiological assessments.

The study adhered to international ethical guidelines following the Declaration of Helsinki and was approved by the ethics committee at Hospital das Clínicas of the University of São Paulo (protocol SDC: 3485/10/074). All participants provided informed consent in the data collection evaluation.

### 2.2 Survey Questionnaire

A questionnaire was applied to collect data on sociodemographic characteristics, medical and reproductive history, and lifestyle habits. The categorization of the race/skin color variable was determined based on self‐declaration, according to the following options: white, black, brown, and other (indigenous or Asian descendant). Education was considered according to years of study and categorized into elementary, secondary, and college/graduate study. Current smoking status was defined as whether smoking had occurred during the last 6 months. The physical activity level was assessed using the short version of the International Physical Activity Questionnaire (IPAQ), validated for the Brazilian population (Matsudo et al. 2001). Participants were classified as physically active if they engaged in moderate physical activity for at least 150 min per week, vigorous‐intensity activity for at least 75 min per week, or a combination of both (Brasil. Ministério da Saúde 2021). Menopausal status was determined based on the self‐reported cessation of menses; specifically, participants who indicated they no longer menstruated were classified as postmenopausal.

The consumption of alcoholic beverages was assessed using objective questions to examine the frequency of intake (daily, weekly, or occasional), the type of alcoholic beverage (beer, red or white wine, and distilled spirits such as whiskey, cachaça, vodka, and liquor), and the amount consumed based on household measurements. Weekly alcoholic beverages consumption was analyzed as follows: household measures and their equivalents in mL were considered for red and white wine as a glass (120 mL), a cup (200 mL), or a bottle (700 mL); for beer, a can/longneck (350 mL) or a bottle (600 mL); and for distilled spirits, a shot (50 mL). To calculate the amount of ethanol in grams, the average alcohol content of the most common beverage brands on the Brazilian market was used: beer = 5%, wine = 12%, and spirits = 39%. First, the amount reported weekly was multiplied by the equivalent measure in mL. Then, the amount of pure alcohol in mL per week was calculated based on the alcohol concentration of each beverage. Subsequently, the amount of alcohol consumed from any beverage was multiplied by the density of alcohol (0.8) to obtain the total amount of pure ethanol in grams per week. Based on the ethanol variable in g/week, a qualitative variable with three categories was also created: abstainers (0 g of ethanol/week), and alcohol consumers were stratified into two groups based on the median alcohol intake within each sex. The first category (AC1) comprised individuals with lower consumption among drinkers, while the second category (AC2) comprised those with higher consumption.

### 2.3 Biochemical Measurements

Blood collection was performed via venipuncture after a 12‐h fasting period. Triacylglycerols, HDL‐c, fasting glucose, and glycated hemoglobin (HbA1c) levels were measured using standard techniques. Triacylglycerols were assessed through the glycerol‐phosphate oxidase colorimetric method, while HDL‐c was measured using a homogeneous enzymatic colorimetric method without precipitation. Fasting glucose was evaluated using the hexokinase method, and HbA1c was determined through high‐performance liquid chromatography (HPLC). Low‐density lipoprotein cholesterol (LDL‐c) was calculated using the Friedewald equation. Diabetes mellitus (DM) was diagnosed with fasting glucose ≥ 126 mg/dL and/or HbA1c ≥ 6.5% and/or the use of antidiabetic drugs (Dintshi and Kone 2022). The HDL‐c/LDL‐c ratio was calculated by dividing HDL‐c by LDL‐c levels. Hypercholesterolemia was defined as a total cholesterol level ≥ 190 mg/dL and/or the use of lipid‐lowering drugs (Faludi et al. 2017). Low HDL‐c was defined as an HDL‐c level ≤ 40 mg/dL and/or the use of lipid‐lowering drugs (Faludi et al. 2017). Hypertriglyceridemia was defined as a triacylglycerols level ≥ 150 mg/dL and/or the use of lipid‐lowering drugs (Faludi et al. 2017).

### 2.4 Anthropometric Evaluations

Anthropometric measurements included weight and height. All parameters were obtained according to a standardized protocol (Lohman et al. 1988). Weight was measured using a calibrated digital scale (Filizola) with a maximum capacity of 180 kg and an accuracy of 100 g. Individuals were weighed standing, barefoot, and in light clothing. Height was determined using a stadiometer (Sanny), measured in centimeters with 1‐mm precision. Body mass index (BMI) was calculated as body weight (kg) divided by height squared (m^2^), and obesity was defined as a BMI ≥ 30 kg/m^2^ (WHO 2000).

### 2.5 Blood Pressure (BP) Measurements

BP was measured with an automated oscillometric device (OMRON, OMRON Eletrônica do Brasil Ltda., SP, Brazil) on the left arm after a 5‐min rest in the seated position. Systolic (SBP) and diastolic blood pressures (DBP) were calculated by the average of three measures within a minimal interval of 3 min. Hypertension was defined as a mean SBP ≥ 140 mmHg and/or DBP ≥ 90 mmHg and/or use of antihypertensive drugs (Barroso et al. 2021).

### 2.6 Statistical Analysis

The data are presented as mean and standard deviation or median and interquartile range for continuous variables and as count and percentage for categorical variables. Normality was tested for the main variables using the Kolmogorov–Smirnov test.

Comparisons of general characteristics stratified by sex and HDL‐c level were conducted using Student's *t*‐test for parametric variables and the Mann–Whitney test for nonparametric variables. For comparisons of general characteristics and HDL‐c levels between the alcohol consumption groups, ANOVA was used for parametric variables and the Kruskal‐Wallis test for non‐parametric variables. Finally, the proportions were compared using Pearson's chi‐squared.

To assess whether the effect of obesity on HDL‐c differed by alcohol consumption, interaction terms between obesity and alcohol categories were created. The statistical significance of interaction terms was evaluated using the Wald test within the logistic regression procedure in SPSS. A two‐sided *p*‐value < 0.05 was considered statistically significant.

A multivariate binary logistic regression analysis was employed to investigate the association between alcohol consumption (abstainers were taken as the reference group) and low HDL‐c, stratified by obesity due to the presence of interaction. All analyses were adjusted for age and age^2^, DM, level of physical activity, smoking, triacylglycerols, and use of lipid‐lowering drugs, with the addition of menopausal status for women.

All statistical procedures were conducted with SPSS version 24.0 statistical package (SPSS Inc., Chicago, Illinois, USA).

## 3 Results

The general characteristics of the sample, stratified by sex, are presented in Table 1. The frequency of obesity is higher in women. Nevertheless, the frequency of low HDL‐c and alcohol consumption (g/week) is higher in men. There was no significant difference in the age and race between the groups.

**TABLE 1:** General characteristics of the sample stratified by sex.

Variables	Total	Men	Women	*p*
*n*	2345	931	1414	—
Age, years	46.0 ± 16.4	46.8 ± 17.0	45.5 ± 16.0	0.076
Race, *n* (%)				0.685
White	1719 (73.3)	681 (73.1)	1038 (73.6)	
Black	141 (6.0)	51 (5.5)	90 (6.4)	
Brown	465 (19.8)	191 (20.5)	274 (19.4)	
Other	17 (0.7)	8 (0.9)	9 (0.6)	
Educational attainment, *n* (%)				0.001
Elementary	1353 (57.7)	566 (60.8)	787 (55.8)	
Secondary	707 (30.1)	282 (30.3)	425 (30.1)	
College/graduate study	282 (12.0)	83 (8.9)	199 (14.1)	
Smokers, *n* (%)	284 (12.1)	148 (15.9)	136 (9.6)	0.003
Physically active, *n* (%)	453 (19.3)	249 (26.7)	204 (14.4)	< 0.001
Obesity, *n* (%)	472 (20.1)	130 (14)	342 (24.2)	< 0.001
DM, *n* (%)	310 (13.2)	111 (11.9)	199 (14.1)	0.132
Hypertension, *n* (%)	907 (38.7)	367 (39.4)	540 (38.2)	0.549
High total cholesterol, *n* (%)	1329 (56.7)	495 (53.2)	834 (59.0)	0.005
Hypertriglyceridemia, *n* (%)	735 (31.3)	306 (32.9)	429 (30.3)	0.197
Low HDL‐c, *n* (%)	677 (28.9)	388 (41.7)	289 (20.4)	< 0.001
Lipid‐lowering drugs, *n* (%)	233 (9.9)	72 (3.0)	161 (11.3)	0.004
Ethanol, (g/week)	14.6 ± 49.3	25.8 ± 69.4	7.3 ± 27.0	< 0.001
Ethanol consumption groups, (g/week)				
Abstainer (*n* = 1890)	—	—	—	
AC1 (*n* = 223)	26.2 ± 11.7	29.6 ± 13.1	21.3 ± 6.7	< 0.001
AC2 (*n* = 232)	122.8 ± 104.6	149.8 ± 121.2	85.8 ± 59.3	< 0.001

*Note:* Continuous variables are presented as mean and standard deviation, while categorical variables are presented as absolute value and percentage.

Abbreviations: AC1, first category of alcohol consumption; AC2, second category of alcohol consumption; DM, diabetes.

Table 2 illustrates an analysis of biochemical, anthropometric, clinical and lifestyle variables, stratified according to the consumption of alcohol. It is noteworthy that in both sexes, the group that consumed the greatest quantity of alcohol per week (AC2) demonstrated a lower age, higher HDL‐c levels, a higher proportion of smokers and a lower proportion of diabetics. Moreover, the frequency of individuals with low HDL‐c was found to be lower only in women who consumed the greatest quantity of alcohol (AC2). Regarding the HDL‐c/LDL‐c ratio, significantly higher values were observed in men from the AC2 group compared to abstainers; however, no such significant differences were maintained among women. Furthermore, no significant associations were found between alcohol intake and fasting glucose, total cholesterol, LDL‐c, and triacylglycerols levels in either men or women.

**TABLE 2:** Anthropometric, biochemical, and lifestyle characteristics stratified by alcohol consumption.

Variables	Men				Women			
	Abstainer (*n* = 664)	AC1 (*n* = 133)	AC2 (*n* = 134)	*p*	Abstainer (*n* = 1226)	AC1 (*n* = 90)	AC2 (*n* = 98)	*p*
Age, years	47.9 ± 17.4^a^	47.5 ± 16.9	40.4 ± 13.7	< 0.001	46.2 ± 16.4^a^	42.2 ± 14.9	40.5 ± 10.8	< 0.001
BMI, kg/m^2^	25.1 ± 4.5	25.1 ± 4.5	25.7 ± 4.7	0.364	26.6 ± 5.5	26.5 ± 5.6	26.1 ± 4.8	0.632
Fasting glucose, mg/dL	93 ± 19	93 ± 17	92 ± 15	0.786	94 ± 24	90 ± 14	90 ± 17	0.142
Total cholesterol, mg/dL^b^	190 (53)	202 (53)	199 (57)	0.134	197 (55)	199 (48)	202 (52)	0.667
LDL‐c, mg/dL^b^	121 (48)	127 (47)	123 (47)	0.441	121 (46)	118 (45)	116 (52)	0.865
HDL‐c, mg/dL^b^	41 (11)^a^, ^c^	45 (14)	47 (15)	< 0.001	48 (14)^a^	50 (16)	52 (15)	0.006
Triacylglycerols, mg/dL^b^	114 (78)	126 (67)	129 (86)	0.117	118 (71)	118 (59)	123 (72)	0.342
HDL‐c/LDL‐c ratio^b^	0.35 (0.16)^a^	0.36 (0.17)	0.37 (0.22)	0.027	0.40 (0.20)	0.44 (0.24)	0.45 (0.24)	0.145
Ethanol, g/week	0^a^, ^c^	29.6 ± 13.1^a^	149.8.4 ± 121.2	< 0.001	0^a^, ^c^	21.3 ± 6.7^a^	85.8 ± 59.3	< 0.001
Low‐HDL‐c, *n* (%)	306 (46.1)	42 (31.6)	40 (29.9)	< 0.001	260 (21.2)	15 (16.7)	14 (14.3)	0.173
Lipid‐lowering drugs, *n* (%)	46 (6.9)	13 (9.8)	13 (9.7)	0.352	148 (12.1)	8 (8.9)	5 (5.1)	0.081
Obesity, *n* (%)	89 (13.4)	15 (11.3)	26 (19.4)	0.118	301 (24.6)	19 (21.1)	22 (22.4)	0.700
DM, *n* (%)	80 (12.0)	23 (17.3)	8 (6.0)	0.017	185 (15.1)	10 (11.1)	4 (4.1)	0.007
Smokers, *n* (%)	79 (11.9)	21 (15.9)	36 (26.9)	< 0.001	108 (8.8)	16 (17.8)	24 (24.5)	< 0.001
Physically active, *n* (%)	167 (25.2)	37 (27.8)	45 (33.6)	0.126	167 (13.6)	19 (21.1)	18 (18.4)	0.078

*Note:* Continuous variables are presented as mean and standard deviation and categorical variables as percentages.

Abbreviations: AC1, first category of alcohol consumption; AC2, second category of alcohol consumption; BMI, body mass index; DM, diabetes.

^a^ Versus AC2.

^b^ Variables are presented as median and interquartile range.

^c^ Versus AC1.

Table 3 presents a detailed analysis of biochemical, anthropometric, clinical, and lifestyle variables, stratified by sex and HDL‐c level. It is observed that in both sexes, the group with low HDL‐c had a higher proportion of individuals with obesity, higher BMI and triacylglycerols levels, and lower total cholesterol levels. The HDL‐c/LDL‐c ratio was significantly lower in the low HDL‐c group for both men and women. Additionally, alcohol consumption was lower in the group with low HDL‐c, but only in men.

**TABLE 3:** Anthropometric, biochemical, and lifestyle characteristics stratified by HDL‐c status.

Variables	Men			Women		
	Normal‐HDL‐c (*n* = 543)	Low‐HDL‐c (*n* = 388)	*p*	Normal‐HDL‐c (*n* = 1125)	Low‐HDL‐c (*n* = 289)	*p*
Age, years	46.8 ± 17.5	46.8 ± 16.4	0.985	45.0 ± 16.1	47.5 ± 15.6	0.018
BMI, kg/m^2^	24.4 ± 4.4	26.2 ± 4.5	< 0.001	26.4 ± 5.4	27.4 ± 5.6	0.007
Fasting glucose, mg/dL	92 ± 18	94 ± 17	0.190	93 ± 23	95 ± 26	0.071
Total cholesterol, mg/dL^a^	198 (57)	187 (54)	< 0.001	199 (53)	186 (53)	< 0.001
LDL‐c, mg/dL^a^	124 (49)	120 (45)	0.049	121 (48)	117 (46)	0.702
HDL‐c, mg/dL^a^	48 (10)	36 (5)	< 0.001	51 (13)	37 (5)	< 0.001
Triacylglycerols, mg/dL^a^	103 (64)	141 (94)	< 0.001	115 (67)	143 (89)	< 0.001
HDL‐c/LDL‐c ratio^a^	0.40 (0.20)	0.30 (0.12)	< 0.001	0.44 (0.21)	0.30 (0.12)	< 0.001
Ethanol, (g/week)	31.6 ± 79	17.7 ± 52.2	0.003	7.8 ± 27.7	5.3 ± 24.0	0.163
Lipid‐lowering drugs, *n* (%)	42 (7.7)	30 (7.7)	0.992	129 (11.5)	32 (11.1)	0.873
Obesity, *n* (%)	59 (10.9)	71 (18.3)	0.001	259 (23.0)	83 (28.7)	0.044
DM, *n* (%)	68 (12.5)	43 (11.1)	0.504	160 (14.2)	39 (13.5)	0.751
Smokers, *n* (%)	81 (14.9)	55 (14.2)	0.743	98 (8.7)	50 (17.4)	< 0.001
Physically active, *n* (%)	163 (30)	86 (22.2)	0.008	163 (14.5)	41 (14.2)	0.913

*Note:* Continuous variables are presented as mean and standard deviation and categorical variables as percentages.

Abbreviations: BMI, body mass index; DM, diabetes.

^a^ Variables are presented as median and interquartile range.

**FIGURE 1:**
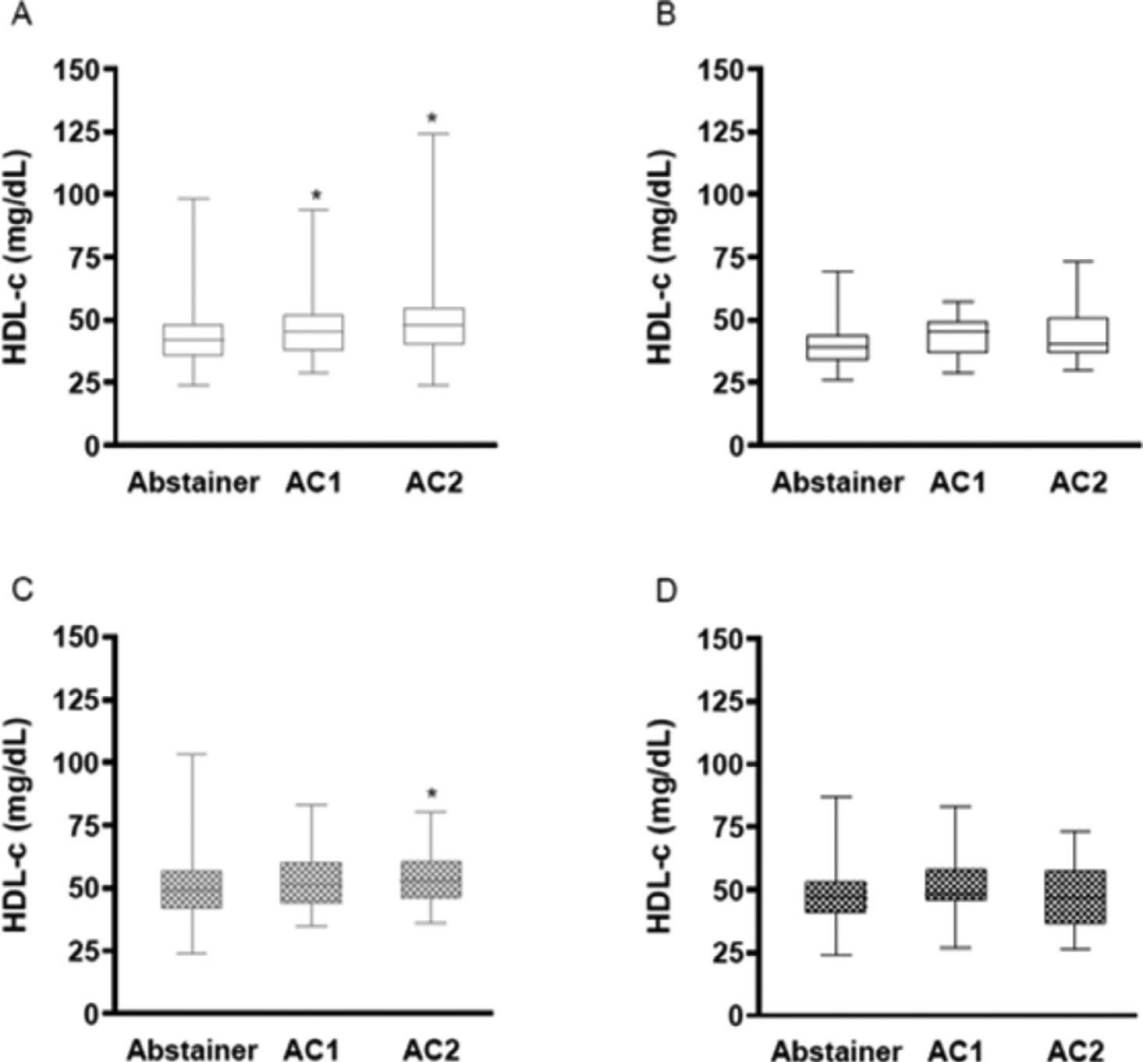
The association between alcohol consumption and HDL‐c levels in individuals with and without obesity. (A) Men without obesity: Gray, unfilled boxes and whiskers; (B) men with obesity: Black, unfilled boxes and whiskers (C) women without obesity: Gray, hatched boxes and whiskers (D) women with obesity: Black, hatched boxes and whiskers. *p < 0.05 versus abstainer group.

Figure 1 shows the association between alcohol consumption and HDL‐c levels in individuals with and without obesity. Note that in the group without obesity, those who consumed more alcohol (AC2) had higher levels of HDL‐c in both sexes (1‐A and 1‐C). However, no difference was found in the group with obesity (1‐B and 1‐D).

A logistic regression analysis was conducted to evaluate the interaction between obesity and alcohol consumption in relation to the odds of having low HDL‐c levels. A statistically significant interaction was found between obesity and the higher category of alcohol consumption (*β* = 0.90, *p* = 0.015), indicating that the effect of alcohol intake on HDL‐c levels is modified by obesity status (data not shown). In Table 4, the multivariate logistic regression analysis demonstrated that, among those without obesity, individuals who consumed more alcohol (AC2) exhibited decreased odds for having low HDL‐c when compared to abstainers. In women, a higher alcohol consumption was associated with a 65% reduction in the odds of having low HDL‐c, while in men, the reduction was approximately 66%. However, no association was observed for individuals with obesity.

**TABLE 4:** Association between alcohol consumption and low HDL cholesterol stratified by obesity status.

Variables	Low HDL cholesterol	
	OR (95% CI), *p*	
	Men	Women
Without obesity		
Abstainer	1	1
AC1	0.508 (0.324–0.796), **0.003**	0.672 (0.335–1.348), 0.263
AC2	0.333 (0.198–0.558), **< 0.001**	0.335 (0.151–0.740), **0.007**
With obesity		
Abstainer	1	1
AC1	0.465 (0.136–1.594), 0.223	0.668 (0.203–2.197), 0.507
AC2	0.476 (0.179–1.262), 0.136	0.638 (0.223–1.830), 0.403

*Note:* For men, the model was adjusted for age and age^2^, diabetes, smoking, physical activity, triacylglycerols, and lipid‐lowering drugs. For women, the model was adjusted for age and age^2^, diabetes, smoking, physical activity, triacylglycerols, lipid‐lowering drugs, and menopausal status. Bold values indicate results that reached statistical significance (*p* < 0.05).

Abbreviations: AC1, first category of alcohol consumption; AC2, second category of alcohol consumption; CI, confidence interval; OR, odds ratio.

## 4 Discussion

The main finding of the present study was the association between alcohol consumption and HDL‐c levels in individuals without obesity, with the highest HDL‐c levels observed among those consuming more alcohol (AC2). Moreover, individuals without obesity who consumed more alcohol (AC2) exhibited a decreased odd for having low HDL‐c when compared to those who abstained. However, no association was observed for individuals with obesity.

Over the past few decades, numerous studies have consistently demonstrated a strong association between low HDL‐c levels and an elevated risk of CVD (Gordon et al. 1989; Badia et al. 2023; Freitas et al. 2009; Castelli et al. 1977; Mora et al. 2013; Assmann et al. 1996). Data from the Framingham Heart Study indicate that a 1 mg/dL increase in HDL‐c corresponds to significant reductions in CVD mortality, with a 3.7% decrease in men and a 4.7% decrease in women (Gordon et al. 1989). However, over recent years, studies have suggested that excessively high HDL‐c levels may be associated with increased cardiovascular risk and all‐cause mortality (Mamede et al. 2024; Madsen et al. 2017), challenging the long‐held view that higher HDL‐c concentrations are uniformly protective. The literature consistently supports the notion that specific behaviors, including light to moderate alcohol consumption, may have a favorable impact on HDL‐c levels. Naud et al. (2020), analyzing data from 4976 participants in the ELSA‐Brasil cohort, reported that alcohol consumption—independent of the type of alcoholic beverage—was significantly associated with increased concentrations of total HDL‐c, HDL_2_, and HDL_3_ subfractions in both women and men. In a case–control study involving 680 individuals of both sexes, Gaziano et al. (1993) reported an inverse association between alcohol consumption and the risk of myocardial infarction. The authors hypothesized that this protective effect may be partially mediated by HDL‐C, as higher alcohol intake was associated with elevated HDL‐C levels. Supporting these observations, der Van Gaag et al. (2001) demonstrated that moderate alcohol consumption over a 3‐week period significantly enhanced cellular cholesterol efflux and plasma cholesterol esterification rates, accompanied by increases in HDL‐c, apolipoprotein AI (apoA‐I), and HDL‐associated phospholipids. These findings suggest that alcohol intake may favorably influence the initial phases of reverse cholesterol transport, irrespective of the specific type of alcoholic beverage consumed.

In our study, individuals in the AC2 group (approximately 17 g/day for men and 12 g/day for women) exhibited higher serum HDL‐c concentrations and lower odds of reduced HDL‐c levels among participants without obesity. This association was not observed in participants with obesity, suggesting that excess adiposity may attenuate the possible beneficial effects of alcohol on lipid metabolism. Additionally, only nine participants presented HDL‐c levels above 90 mg/dL, which restricted analysis of the association between alcohol consumption and very high HDL‐c concentrations. In line with our findings, Fricker et al. (1990), in a cohort of 653 women treated at the Nutrition Clinic of Bichat Hospital, reported that moderate alcohol intake (approximately 10 g/day) was associated with increased HDL‐c concentrations in women without obesity. This effect, however, was absent among individuals with obesity, possibly due to impaired lipolytic activity associated with excess adiposity. Further supporting this association, Hagiage et al. (1992) investigated the HDL‐c response to alcohol consumption (30 g/day for 14 days) in normolipidemic men with obesity compared with normal‐weight controls. Participants with obesity exhibited markedly lower HDL‐c concentrations, approximately 50% lower than those of controls, primarily due to a reduction in the HDL_2_ subfraction, while HDL_3_ levels remained comparable between groups. Following alcohol intake, only the control group showed an increase in apoA‐I, suggesting that obesity may blunt the HDL‐c metabolic response to ethanol, possibly through enhanced turnover of the HDL_2_ fraction and increased influence of adipose tissue on HDL‐c kinetics.

Regarding the specific type of beverage, while wine has been traditionally highlighted for its polyphenol content, recent evidence from systematic reviews and meta‐analyses suggests that the lipid‐modulating effects are largely consistent across different alcoholic beverages. A recent meta‐analysis of clinical trials confirmed that wine consumption significantly increases HDL‐c and apolipoprotein A‐I (ApoA‐I) levels (Lucerón‐Lucas‐Torres et al. 2025). Similarly, beer consumption has also been associated with favorable changes in HDL‐c and vascular protection, suggesting that its moderate intake does not adversely affect the lipid profile (Spaggari et al. 2020). Supporting the hypothesis that the ethanol component itself is the primary driver of these changes, several meta‐analyses of intervention studies have demonstrated that moderate alcohol intake—regardless of the source—consistently increases HDL‐c and ApoA‐I concentrations (Rimm et al. 1999; Brien et al. 2011; Huang et al. 2017; Khatiwada et al. 2025). These findings suggest that the increase in HDL‐c observed in our study is likely mediated by the ethanol content predominant in beer, the choice of 79% of our alcohol‐consuming participants, rather than nonalcoholic compounds specific to wine.

Several physiological mechanisms may explain the association between moderate alcohol consumption and higher HDL‐c levels observed in individuals without obesity. One possible explanation is that alcohol stimulates hepatic production of apoA‐I, a key structural component of HDL‐c, promoting reverse cholesterol transport (Badia et al. 2023; Naud et al. 2020; Brinton 2012). This is supported by comprehensive metabolic analyses, which demonstrate that alcohol consumption induces a complex signature characterized by increased lipid concentrations across HDL subclasses and adverse reductions in LDL particle size (Würtz et al. 2016). Additionally, alcohol consumption has been linked to increased lipoprotein lipase (LPL) activity, an enzyme responsible for triacylglycerols hydrolysis in lipoproteins, which may enhance HDL‐c formation (Naud et al. 2020; Nishiwaki et al. 1994). Another potential mechanism involves the upregulation of cholesterol efflux pathways, particularly through ATP‐binding cassette transporters (ABCA1 and ABCG1), facilitating cholesterol transfer from macrophages to HDL‐c particles (Badia et al. 2023; Hoang et al. 2008). Moreover, alcohol may exert anti‐inflammatory and antioxidant effects, reducing oxidative stress and preserving HDL‐c functionality (Rader and Hovingh 2014; Mineo and Shaul 2012). However, in individuals with obesity, these benefits may be attenuated by reduced adiponectin levels (Cnop et al. 2003) and the presence of other metabolic disturbances, such as insulin resistance (Rashid and Genest 2007), which impair HDL‐c metabolism and reverse cholesterol transport (Shah et al. 2001).

This study has some limitations. First, all participants reside in a small rural town in Southeast Brazil, which may limit the generalization of the findings to the broader Brazilian population. Second, given the cross‐sectional design, causal inferences cannot be made. As this is a cross‐sectional study, residual confounding cannot be ruled out. Another limitation of this study is the lack of segregation by alcoholic beverage type. While wine contains nonalcoholic components like polyphenols that may exert additional antioxidant effects, the small number of wine consumers in our cohort (10%) limited the statistical power for a separate analysis. It is important to note, however, that beer was the beverage of choice for 79% of the alcohol consumers in this study. This pattern is consistent with the socioeconomic profile of the region, where wine is less accessible, and suggests that our results mainly represent the impact of beer consumption on the studied outcomes. Nonetheless, the study's primary strengths include an adequately large sample size, which ensures robust statistical power. Furthermore, HDL‐c levels were assessed using a standardized method commonly employed in clinical practice, and the alcohol consumption questionnaire was administered by a single, thoroughly trained interviewer.

In conclusion, moderate alcohol consumption, observed in the AC2 group, was associated with higher HDL‐c concentrations and lower odds of low HDL‐c, particularly among individuals without obesity. These findings contribute to the understanding of the complex interplay between alcohol intake, lipid metabolism, and adiposity. However, considering the well‐established association between alcohol use and a range of diseases, these results should not be interpreted as an endorsement of alcohol consumption. In line with current World Health Organization guidelines, which recommend zero alcohol consumption to minimize health risks, the promotion of alcohol intake for cardiovascular or metabolic benefit is not supported from a public health perspective.

## Author Contributions

L.E.B.C., D.Z., and J.H.S. analyzed the data, interpreted the results, and drafted the manuscript. R.O.A. and Oscar Geovanny Enriquez‐Martinez participated in the design of the work and contributed to the analysis of data and interpretation of results. A.C.P. and C.M.O. contributed to acquisition of data. R.O.A. and J.H.S. participated in the design of the work and supervised the acquisition of data. R.O.A. conceptualized and designed the study, and critically reviewed the manuscript for important intellectual content. All authors approved the final manuscript as submitted and agree to be accountable for all aspects of the work.

## Funding

This work was supported by the Hospital Samaritano Society (25000.180.664/2011‐35).

## Conflicts of Interest

The authors declare no conflicts of interest.

## Data Availability

The data that support the findings of this study are available from the corresponding author upon reasonable request.
